# Therapeutic Whole Lung Lavage for Pulmonary Alveolar Proteinosis: Technique With Continuous Video‐Enabled Double Lumen Endotracheal Tube

**DOI:** 10.1002/rcr2.70153

**Published:** 2025-03-18

**Authors:** Kevin Davidson

**Affiliations:** ^1^ Pulmonary and Critical Care WakeMed Health & Hospitals Raleigh North Carolina USA

**Keywords:** alveolar, bronchoalveolar, lavage, proteinosis, pulmonary

## Abstract

Therapeutic whole lung lavage with a continuous video‐enabled double‐lumen endotracheal tube improves the safety of therapeutic whole lung lavage in autoimmune pulmonary alveolar proteinosis.

## Graphical abstract

Autoimmune pulmonary alveolar proteinosis is a rare lung disease which causes dyspnoea, cough, and subsequent respiratory failure due to the accumulation of surfactant proteins in the lung. Using a continuous video‐enabled double‐lumen endotracheal tube, we demonstrate how this procedure can be done with significantly improved patient safety.

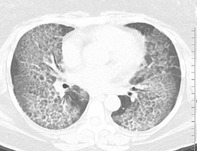

## 1 Case Video

Autoimmune pulmonary alveolar proteinosis is a rare lung disease which causes dyspnoea, cough, and subsequent respiratory failure due to the accumulation of surfactant proteins in the lung [1]. Isolating each lung sequentially under anaesthesia to perform therapeutic whole lung lavage is a proven technique to treat this condition [1, 2]. However, there are substantial risks of performing whole lung lavage including double‐lumen endotracheal tube migration, challenges with isolating each lung for high‐volume irrigation or ventilation, and hypoxemia [1, 2]. Using a continuous video‐enabled double‐lumen endotracheal tube, we demonstrate how this procedure can be done with significantly improved patient safety (Video 1).

**VIDEO 1:** The video highlights the key images in proper double‐lumen endotracheal tube placement during sequential therapeutic whole lung lavage of the left and right lungs. Viewers are shown key technical aspects of this procedure, which is performed for a rare lung disease: pulmonary alveolar proteinosis. The procedure can be life‐saving and is infrequently performed. Patient safety is improved by continuous video monitoring of tube placement throughout the entire procedure to reduce the risk of complications. Video content can be viewed at https://onlinelibrary.wiley.com/doi/10.1002/rcr2.70153

## Author Contributions

Kevin Davidson owns this video, wrote captions and the manuscript, performed edits and entered the submission.

## Ethics Statement

The author declare that appropriate written informed consent was obtained for the publication of this manuscript and accompanying images.

## Conflicts of Interest

The author declares no conflicts of interest.

## Data Availability

Data sharing not applicable to this article as no datasets were generated or analysed during the current study.
